# Project BESPOKE (Integrated Bio-Behavioral Assessment of HIV and STI Among Young Tertiary Student Men Who Have Sex With Men in Nairobi, Kenya): A Respondent-Driven Sampling Survey Protocol

**DOI:** 10.3389/fpubh.2021.619694

**Published:** 2021-10-11

**Authors:** Samuel Waweru Mwaniki, Peter Mwangi Mugo, Thesla Palanee-Phillips

**Affiliations:** ^1^School of Clinical Medicine, Faculty of Health Sciences, University of the Witwatersrand, Johannesburg, South Africa; ^2^University Health Services, University of Nairobi, Nairobi, Kenya; ^3^Kenya Medical Research Institute-Wellcome Trust Research Programme, Nairobi, Kenya; ^4^Wits Reproductive Health and HIV Institute, University of the Witwatersrand, Johannesburg, South Africa

**Keywords:** healthcare providers, HIV, integrated bio-behavioral assessment, Kenya, men who have sex with men, respondent-driven sampling, sexually transmitted infections, young university students

## Abstract

**Introduction:** Globally, men who have sex with men (MSM) are a key population for the human immunodeficiency virus (HIV) epidemic. Among MSM, young men who have sex with men (YMSM) are disproportionately affected by HIV and other sexually transmitted infections (STI). However, there is a dearth of research and interventions targeting HIV/STI prevention among YMSM. In Kenya, there is paucity of knowledge on the burden of HIV/STI and related factors among YMSM, including tertiary student men who have sex with men (TSMSM). The barriers TSMSM experience in accessing and utilizing health services in their learning institutions have seldom been explored. In the context of healthcare providers (HCP) working in tertiary institutions, little is known about their knowledge, attitudes, and practices toward providing services to TSMSM.

**Methods:** The aims of the study are to: estimate prevalence and correlates of HIV/STI among TSMSM; estimate population size of TSMSM; explore experiences of TSMSM with access and utilization of health services; and assess HCP knowledge of, attitudes toward, and practices in provision of services to TSMSM. A mixed-methods approach will be used in three phases: Phase I—formative qualitative research will be conducted to understand TSMSM social networks, select “seeds”, and explore strategies for implementing a respondent-driven sampling (RDS) survey. Interviews will be conducted with at least three staff who work in community based/non-governmental organizations (CBO/NGO) that serve MSM and at least 10 TSMSM. Phase II—an integrated bio-behavioral assessment (IBBA) will be conducted, where 200 TSMSM recruited by RDS will be offered HIV/STI testing, complete a behavioral survey, and provide information for population size estimation (PSE). Phase III—in-depth interviews will be held with 20 TSMSM selected from 200 TSMSM in phase II, to explore their experiences with access and utilization of healthcare services. Focus group discussions (FGD) will be conducted with HCP working in tertiary institutions to assess their knowledge of, attitudes toward, and practices in providing services to TSMSM. Data collection started in September 2020 and is expected to end by September 2021.

**Discussion:** Findings from this study will be useful in informing HIV/STI prevention programming for TSMSM, by policy makers such the Kenyan ministries of health and education, tertiary education institutions, service providers, advocacy groups, and other interested stakeholders.

## Introduction

Despite the global advances made in prevention of human immunodeficiency virus (HIV) infections, men who have sex with men (MSM) continue to experience a disproportionate burden of HIV infections (1). Globally in 2019, close to one quarter (23%) of all new adult HIV infections were among MSM (1). The risk of acquiring HIV is 26 times higher among MSM compared to heterosexual men (2). Data from Sub-Saharan Africa (SSA) countries show high rates of HIV infection among MSM. A recent study in South Africa reported a HIV infection rate of 37.5% (3). High HIV infection rates have also been reported in other Southern African countries such as Namibia−12.4%, Botswana−19.6%, and Malawi−12.5–21.4% (4–6). Similarly, this pattern is observed in West African countries where HIV infection rates are also high in countries such as Nigeria−34.9% (7), Ivory Coast−18% (8), and Ghana−17% (9). In the Eastern Africa region, high rates of HIV infection have also been reported in Tanzania−30.2% (10), Uganda−12.9% (11), and Kenya−26.4% (12). Furthermore, high rates of various sexually transmitted infections (STI) among MSM have been reported in various countries in SSA. These include: *Chlamydia trachomatis* (CT) in Tanzania−7.5% (10) and Kenya−26% (13); *Mycoplasma genitalium* (MG) in Nigeria−36.8% (14); *Neisseria gonorrhoae* (NG) in Tanzania−14.4% (10) and Kenya−26% (13); *Treponema pallidum* (TP) in Malawi−12.3% (6) and Uganda−9% (11); and *Trichomonas vaginalis* (TV) in South Africa−9% (15).

Globally, the HIV epidemic remains poorly defined among young men who have sex with men (YMSM) aged 15–24 years. There is minimal data on population size estimates of YMSM, their HIV rates as well as risk and protective factors. This, in part, is attributed to lack of research and surveillance, and the difficulty of reaching YMSM who may fear disclosing their same-sex behavior due to social stigma (16). Social stigma is common among MSM across SSA, and may further influence risks for HIV and STI via its association with depression (17, 18). In most countries, YMSM are made especially vulnerable to HIV by criminalization of homosexuality, widespread discrimination, stigma, and violence, power imbalances in relationships and, sometimes, alienation from family and friends (19). Overall, YMSM are often at greater risk of acquiring HIV than young heterosexual males or older MSM (20).

Notwithstanding the relative paucity of research targeting YMSM in the global context, estimates of HIV prevalence among MSM have pointed to consistently high rates of infection among YMSM (21). A meta-analysis of studies published between 2006 and 2012 in China estimated the prevalence rates of HIV and TP among high school and college YMSM students to be 4.4 and 5.7%, with separate study prevalence rates ranging from 1.1 to 26.9% and 2.2 to 15.4%, respectively (22). In the USA, between 2008 and 2010, new HIV infections among YMSM aged 13–24 years increased by 22%, compared to 12% among MSM overall (23). Also in the USA, in 2011, 92.8% of all diagnosed HIV infections among young males aged 13–19 years was attributed to YMSM (24). In low and middle income countries, HIV prevalence rates as high as 9.6% in Nigeria, 12.7% in Senegal, 24% in Bahamas, and 28% in Jamaica, have also been reported among YMSM (25).

Among tertiary students in the USA, TSMSM were found to be especially vulnerable to HIV infection (26). Further, a study of TSMSM attending university health services in the USA showed that 3% of participants had either a rectal CT or pharyngeal NG infection (27). Similarly, a study among university students in China demonstrated that TSMSM were more sexually active and at greater risk of STI than their non-TSMSM counterparts (28). Additional studies in China have reported considerable rates of HIV and TP among TSMSM, ranging from 2.5 to 5.5 and 4.4 to 7%, respectively (29–31), with many TSMSM only learning of their HIV-positive serostatus at university (32). In a South African higher education sector HIV survey, 6% of male students reported engaging in same sex practices and HIV prevalence among TSMSM (4.1%) was greater than twice that of heterosexual male students (1.7%) (33).

Tertiary student men who have sex with men like other YMSM engage in high risk behavior. Upon joining tertiary institutions, they are suddenly exposed to peers with similar sexual orientation, more freedom, and more socializing opportunities. Subsequently, many have sex more frequently, and some might have unprotected anal intercourse (UAI) more frequently (32). A meta-analysis of studies among Chinese high school and university student MSM found high rates of UAI, unsatisfactory condom use during the last homosexual anal intercourse, and having multiple sex partners (34). Additionally, TSMSM in the USA have been shown to have complex and expansive sexual networks within which HIV/STI may circulate widely and amplify epidemics (35). The results of a behavioral survey in 14 higher education institutions in South Africa indicated a high prevalence of various risk factors for HIV/STI transmission among TSMSM, including: high partner turnover, concurrent sexual partners, presence of STI, early sexual debut, having female sex partners, forced sex experiences, inconsistent condom usage, and alcohol and drug use (36). With most tertiary students owning smart phones, TSMSM like other MSM may also use geosocial applications such as Grindr® in seeking high risk casual sex partners online (36, 37).

In Kenya, there is a dearth of knowledge on the burden of HIV among YMSM, including TSMSM. Additionally, the prevalence of STI in TSMSM is also not known, despite the fact that untreated STI biologically potentiate transmission and acquisition of HIV (38–42), as well as cause considerable morbidity on their own (43). Epidemiological data of related indexes such as HIV/STI-related knowledge and risk factors among TSMSM is also sparse. As well, data on population size estimation (PSE) of TSMSM to inform advocacy activities, resource allocation, and scale of requisite prevention/treatment programs (44), is not available. Besides, the barriers TSMSM experience in accessing and utilizing general and sexual health services in their academic institutions have also not been explored. Further, in the context of healthcare providers (HCP) working in tertiary institutions, little is known about their knowledge of, attitudes toward, and practices in provision of services to TSMSM—factors which are critical to access and utilization of health services by the TSMSM. These are the knowledge gaps this study aims to fill.

## Methods and Analysis

### Aim

The primary aim of this study is to estimate the prevalence of HIV and STI (CT, MG, NG, TP, and TV) and their correlates (such as unprotected sexual intercourse, multiple sexual partners, alcohol, and drug use) among TSMSM. The secondary aims are to: estimate the population size of TSMSM; explore the experiences of TSMSM with access and utilization of health services; and assess tertiary institutions HCP's knowledge of, attitudes toward, and practices in provision of services to TSMSM.

### Study Design

**Figure 1:**
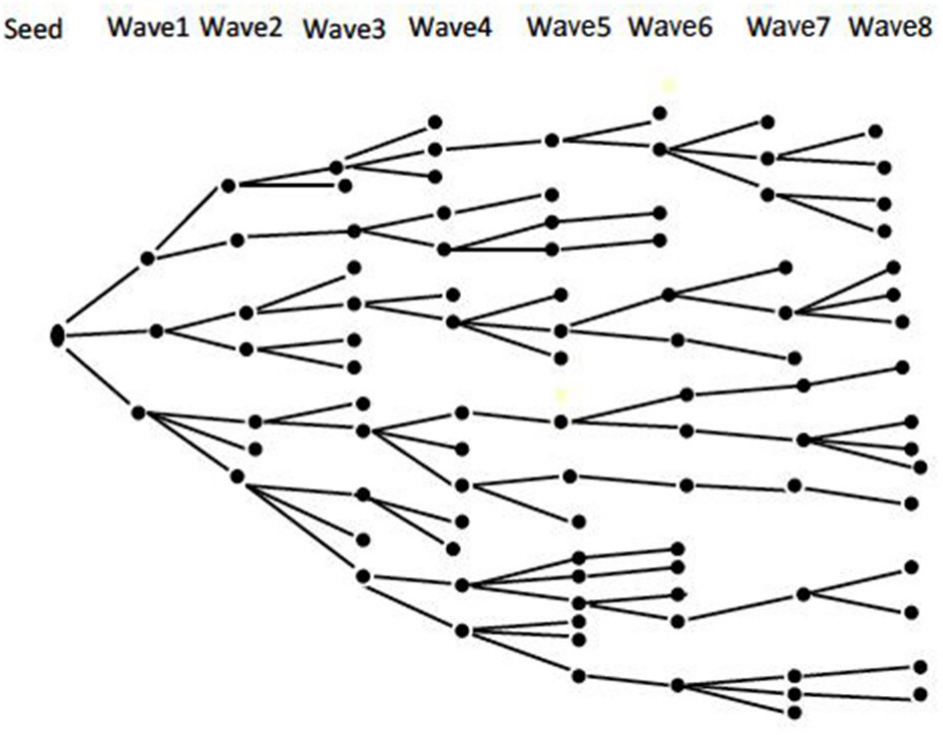
Theoretical RDS recruitment chain [Source—Open Access: Johnston and Sabin, (49)].

An integrated bio-behavioral assessment (IBBA) of HIV and STI will be conducted among TSMSM recruited through the respondent-driven sampling (RDS) method. Integrated bio-behavioral assessment is a term that refers to an overarching approach to tracking HIV and STI prevalence—biological component, and linking these data with related factors—behavioral component, among key populations at higher risk for HIV/STI infection, such as MSM (45). Respondent-driven sampling is both a recruitment and statistical method (46). It utilizes “snowball sampling” where individuals recruit those they know, the recruited individuals in turn recruit those they know and so on. This recruitment is combined with a mathematical model that weights the sample to compensate for the non-random (snowball) sampling (46). Using RDS makes it possible to draw statistically valid samples from hidden populations—such as MSM (47), and provide unbiased population estimates (such as disease prevalence) and measures of the precision of those estimates (such as confidence intervals) (48). Recruitment begins with “seeds” purposely selected from the target population and given a limited number of “coupons” to recruit their peers into the study. Participants who are recruited by the “seeds” form the first “wave” of recruitment. Participants in the first “wave” are then issued with “coupons” to recruit the second “wave” of participants. This process goes on until the desired sample size is reached. Figure 1 shows a theoretical RDS recruitment chain (49).

As per guidelines (50), supplemental studies will be incorporated into the IBBA. Prior to the IBBA, formative research in form of semi-structured interviews will be conducted with service providers who work in community based/non-governmental organizations (CBO/NGO) that serve MSM; and TSMSM referred to the study team by the service providers. The aims of the formative research are to: understand the social networks of TSMSM, assess the appropriateness and acceptability of using RDS, inform survey logistics and help with the recruitment of seeds. During the IBBA, data will also be collected to conduct PSE for TSMSM. In addition, qualitative in-depth interviews will be conducted with selected TSMSM to explore their experiences with access and utilization of health services. Finally, focus group discussions (FGD) will be held with HCP from tertiary institutions to assess their knowledge of, attitudes toward, and practices in provision of services to TSMSM. Standard operating procedures will be developed and used for the various components of the study.

### Study Setting

**Figure 2:**
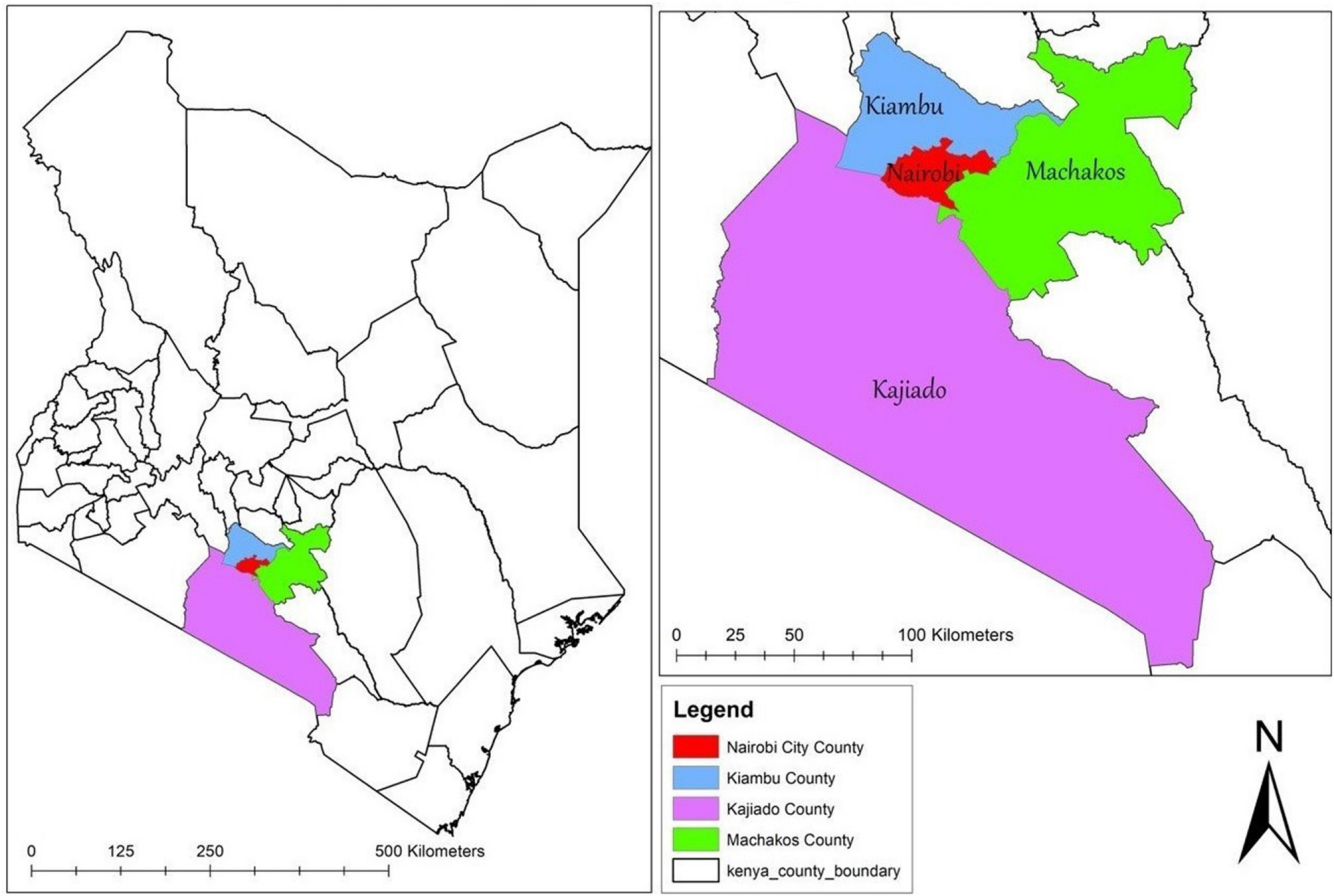
Map showing the study catchment area. The map was created by the author by downloading shapefiles from World Resource Institute (https://www.wri.org/resources/data-sets/kenya-gis-data)—an open source website. The data was then exported to ArcGIS V.10.5 (https://desktop.arcgis.com/en/)—a free mapping, analysis and data storage system used for creating and sharing maps and other geographic content, to create the current map.

The study will be conducted in Nairobi, Kenya. Apart from being Kenya's capital, Nairobi is one of the 47 administrative regions (counties) of Kenya. Based on what has been observed in other places where data is available (35), it is assumed that the TSMSM community in Nairobi is networked to some extent. As a result, the study will aim to reach TSMSM in tertiary institutions that are in the larger Nairobi metropolitan area, that includes tertiary institutions within a radius of approximately 25 km (16 miles) from the Nairobi central business district. This area extends to the neighboring counties of Kiambu, Kajiado, and Machakos as shown in Figure 2.

Research with TSMSM will be conducted in a study office within Nairobi's central business district. The study office had successfully been used to recruit 618 MSM participants for another study among MSM in 2017. It is easily accessible through various public transport routes used by TSMSM from different institutions around the city. In addition, it offers privacy to study participants and has no identifiers that would expose participants as being MSM. It is owned and will be offered to the study team at no cost by Partners in Health and Development in Africa (PHDA)—a non-profit organization affiliated to the University of Nairobi and University of Manitoba—that conducts HIV/STI research among key populations, including MSM. Human immunodeficiency virus testing will be conducted at the study office. Sexually transmitted infections sample storage and testing will be conducted at the Connect Afya Medylinks laboratories. Research with service providers working in CBO/NGO that serve MSM, and HCP from tertiary institutions will be conducted at their work places or the site of the IBBA or any other agreed location, whichever place is most convenient for them.

### Characteristics of Participants

The study population will be TSMSM studying and staying within Nairobi; staff working in CBO/NGO that serve MSM in Nairobi; and HCP working in tertiary institutions.

#### Service Providers Working in CBO/NGO That Serve MSM

Service providers will be eligible to participate if they: directly work with MSM and provide written informed consent to participate in the study. They will be ineligible if they are unable or unwilling to provide written informed consent to participate.

#### TSMSM

Tertiary student men who have sex with men will be eligible to participate if they: are at least 18 years of age; registered students in a tertiary institution (college/university) in Nairobi; were assigned male sex at birth (transgender women will be eligible if they have had anal sex with a man in the last 12 months); have had consensual receptive or insertive oral or anal sexual intercourse with an adult man (18 years and above) in the last 12 months; provide written informed consent to participate in the study; and have a valid recruitment coupon (except for seeds). Tertiary student men who have sex with men will be ineligible for the study if they: are below 18 years of age; have not had consensual receptive or insertive oral or anal sexual intercourse with an adult man in the last 12 months (only having had vaginal intercourse with a postoperative transgender woman will not constitute eligibility); do not have a valid recruitment coupon; have participated in the study before; are unable or unwilling to provide written informed consent in the language available at the study site; and have any condition that in the opinion of the investigators will interfere with successful completion of study procedures.

Because of cash reimbursements given for participation in the study, participants may want to take part more than once by forging coupons or recruit participants who do not meet the eligibility criteria. To minimize this, the design of the coupon will incorporate a unique feature such as a mark which is not easily visible but can be identified by a trained member of the study team. A biometric system in form of a fingerprint reader—DigitalPersona™ (51) will be used to deter double participation. As well, prior to recruitment into the study, the study team will screen participants using a set criterion. Under this criterion, participants will be requested to show their student identification documents. The screening staff will compare the image on the identification document with the prospective participant's facial appearance and upon verification, return the document to the prospective participant. Copies of the identification documents will not be requested for retention by the study team.

#### HCP Working in Tertiary Institutions

Healthcare providers will be sampled from the following cadres: health records information officers (usually working as front office staff in most health centers in tertiary institutions and have first contact with the students seeking services), nurses, clinicians, pharmacists, laboratory technologists, and counselors. They will be eligible to participate if they: are professionally trained and licensed to practice in their respective fields; directly provide services to students in tertiary institutions; and provide written informed consent to participate in the study. They will be ineligible to participate in the study if they: do not directly provide services to students (e.g., those who only hold administrative positions) and are unable or unwilling to provide written informed consent to participate.

### Sampling Procedures and Sample Size Determination

#### Phase I: Formative Research

At least one service provider will be purposely selected from each of the two CBO that offer MSM-exclusive health services and one NGO that in addition to MSM, also serves other key populations. After participation, these providers will help with the identification of TSMSM who will also participate in the formative phase. These providers may be or not be HCP. The providers will be given the contacts of the principal investigator (PI) and asked to request TSMSM known to them to contact the PI about possible participation in the formative research. At least 10 TSMSM will be purposely selected to take part in the in-depth interviews. These TSMSM will ideally be peer leaders, each with a social network size of at least nine people, be interested in the study and helping the TSMSM community. The “seeds” for the RDS will be drawn from these interviewees, upon completion of the formative research. Since this phase is formative and is intended to understand the social networks of TSMSM, explore the logistics of conducting the RDS among TSMSM, as well as selecting “seeds” for the RDS, a sample of 10 was deemed adequate to enable selection of five seeds and retain the other five as standby “seeds.” A previous systematic review demonstrated that on average, RDS studies used 10 seeds [range, 2–32, median 8.0, intra-quartile range (IQR) 6.0–13.0] and had 1.6 (range 0–19, median 0, IQR 0–2.0) unsuccessful seeds per study (52). It is analytically desirable to have a small number of seeds and longer recruitment chains that ensure recruitment goes deeper into the networks of the target population, and where this is not practical, additional seeds may be used (53). Since the study seeks to recruit participants from a hidden population within the tertiary institutions, no particular tertiary institution will be targeted for the recruitment of the 10 TSMSM. However, the study team will seek to have diversity among the 10 TSMSM in terms of the type of institutions they attend (public/private) and geographical location of their institutions within Nairobi (Central, North, South, East, or West).

#### Phase II: IBBA and PSE

For the IBBA and PSE, 200 TSMSM will be recruited. The sample size for the IBBA was calculated to estimate the prevalence of HIV among TSMSM, based on World Health Organization's (WHO) 2017 bio-behavioral survey guidelines for populations most at risk for HIV (50). The formula below was used:


$$\begin{array}{l} n\;=\;\frac{DEFF\;*\;Z^{2}\;*\;P\;*\;\left(1-P\right)}{d^{2}} \end{array}$$

where:

*n* = minimum sample size required,
*DEFF* = design effect,
Z = z-score for the desired confidence level (usually 1.96 for 95% confidence),
*P* = expected proportion,
*d* = precision.

Applying a *DEFF* of 3 to account for clustering that occurs as a result of recruitment by RDS, *P* of 4.1% based on HIV prevalence from a previous study among TSMSM in South Africa (33), and a precision of 5%, the minimum sample size required is:


$$\begin{array}{l} n=\;\frac{\left(3\;*\;1.96\;*\;1.96\;*\;0.041\;*\;0.959\right)}{0.05^{2}}=181.3 \end{array}$$

Adding 10% to the sample to account for non-response, the adjusted sample size is:


$$\begin{array}{l} N=181.3\;*\;\frac{110\;}{100}=199.4\;\approx 200 \end{array}$$

#### Phase III: In-depth Interviews With TSMSM and FGD With HCP Working in Tertiary Institutions

##### TSMSM

For the qualitative in-depth interviews, 20 TSMSM (10% of the total 200 TSMSM in the IBBA) will be selected to participate. To recruit participants in an unbiased manner, every 10^th^ participant in the IBBA will be invited to take part in the in-depth interviews. A similar approach was used in a study in Tanga, Tanzania, and none of the participants in the IBBA who were invited to take part in the qualitative in-depth interviews declined (54). Should an invited participant from the IBBA decline to take part in the interviews, the next participant within the next interval of 10 participants will be invited. We expect to get a consenting participant within that interval before getting to the next scheduled 10^th^ participant, from where recruitment for the qualitative in-depth interviews will continue as initially scheduled.

##### HCP Working in Tertiary Institutions

Tertiary institutions that have students' health centers will be identified. Healthcare providers from these institutions will be purposively selected to participate in the focus groups. At least one HCP will be selected from each tertiary institution per the various cadres outlined under the eligibility criteria. A focus group will consist 6–8 HCPs from one cadre to ensure homogeneity, minimize the effects of group power dynamics, and encourage more openness during discussions. One focus group will be held for every cadre, which translates to six focus groups in total. Previous research has shown that the most prevalent themes within a dataset can be identified with only three focus groups (55). As such, the six focus groups proposed in this study are deemed adequate to bring out prevalent themes from the topics of discussion. Focus groups were selected for their usefulness in exploratory research in under-researched topics (56), such as provision of services to TSMSM in this case. They are also effective when participants do not have easily accessible ways of constructively talking about a research topic (57), as is the case with debates on same-sex behavior which are usually hostile in SSA countries such as Kenya (58). Focus groups create a setting where different perceptions, judgments, and experiences about a certain topic come up. Participants are then stimulated to air their own views, by what they hear other members of the group say, thus providing deeper and detailed information on the research topic being discussed (59).

### Data Collection

#### Phase I: Formative Research

The data collected from this phase will be used to inform the implementation of the second phase of the study. Formative research will entail interviews with at least three staff who work in CBO/NGO that serve MSM and at least 10 TSMSM from tertiary institutions in Nairobi, Kenya. The interview guides that will be used in this phase were modified for this study from the Integrated HIV Bio-behavioral Surveillance Toolbox by the University of California, San Francisco (60).

#### Phase II: IBBA and PSE

##### IBBA

The data collected in this phase will be used to reach the first aim of the study. An IBBA will be conducted to collect data from 200 TSMSM recruited through the RDS method. The recruitment will start with five “seeds” selected from the TSMSM interviewed in phase I. The recruitment will continue in “waves” until the calculated sample size is reached. The behavioral assessment will be conducted by self-interview on tablets using questionnaires set up on REDCap® software (Vanderbilt University). As and when needed, a member of the study team will be at hand to guide participants with navigating the self-interviews. The behavioral assessment will collect data on the following themes: demographic information; use of smart phone apps and social media; sexual behavior history; last sex partner matrix; condom access and use; lubricant access and use; rectal douching and enema use; HIV testing history; HIV self-testing; HIV care and treatment; pre-exposure and post-exposure knowledge and use; STI; healthcare services use; stigma, discrimination, and violence; alcohol, injection, and other drugs' use; and mental health assessment (anxiety, depression, and stress).

After completing the behavioral assessment, participants will be offered tests for HIV, CT, MG, NG, TP, and TV. Venepuncture will be performed by a trained HCP to collect 4–5 ml of blood for HIV and TP screening and confirmatory tests. Participants will be given instructions on how to collect approximately 20 ml of first void urine for CT, MG, NG, and TV testing. Anorectal and pharyngeal swabs for CT, MG, NG, and TV testing will be collected by a different trained HCP. Participants will be free to choose the gender of the HCP who will collect the anorectal swab. Alternatively, participants will be given instructions on how to collect the anorectal swab if they are not comfortable with the HCP doing it.

Human immunodeficiency virus and TP testing will be done using rapid diagnostic tests as provided for by Kenya national and WHO testing algorithms. CT, MG, NG, and TV testing will be done using commercial nucleic acid amplification tests.

Pre- and post-testing counseling will be offered to all participants. Participants who test positive for HIV and are not on care will be referred to facilities of their choice for HIV care and treatment. Participants who test negative for HIV and are at high risk for HIV infection will be referred for pre-exposure prophylaxis (PrEP) if not already on PrEP. The referral will be done by the counselor or a clinician (clinical officer or nurse) who is a member of the study team at the PHDA study office. Participants who test positive for CT, MG, NG, TP, and TV will be offered treatment based on Kenyan treatment guidelines.

For participants who consent to this, leftover blood will be stored for future tests. These tests may include: HIV viral load if the test result for HIV is positive; CD4 cell count if the test result for HIV is positive; Hepatitis B Virus (HBV) and Hepatitis C Virus (HCV). Approximately 2–3 ml of serum will be extracted from the left-over blood. This will be stored for a maximum of five years in the biobank at Kenya AIDS Vaccine Initiative—Institute of Clinical Research, University of Nairobi. No further research will be carried out on these specimens without ethical clearance from the relevant research and ethics committees i.e., University of the Witwatersrand, Human Research Ethics Committee-Medical, and Kenyatta National Hospital—University of Nairobi Ethics and Research Committee.

The questionnaire that will be used in the behavioral assessment was developed for this study, with modifications from various sources, including: Integrated HIV Bio-behavioral Surveillance Toolbox by University of California, San Francisco (60), Rectal Douching and Enema Survey by International Rectal Microbicide Advocates—University of California, Los Angeles (61), MSM Stigma Metrics by Johns Hopkins University (62), and American Men's Internet Survey by Emory University (63). In addition, the Alcohol Use Disorders Identification Test (AUDIT) (64), Perceived Stress Scale-10 (PSS-10) (65), Patient Health Questionnaire-9 (PHQ-9) (66), and Generalized Anxiety Disorder-7 (GAD-7) (67) tools were included in the IBBA questionnaire. The PSE questionnaire was developed for this study.

##### PSE

Prior to the behavioral self-interview, an interviewer-guided questionnaire will be used to collect data for PSE. A combination of methods embedded within the RDS study will be used to produce multiple estimates of the population sizes of TSMSM. These are: wisdom of the crowds, successive sampling (SS)-RDS and RDS-network based capture recapture.

#### Wisdom of the Crowds

Under this method, participants will be asked to provide their best estimate of the number of TSMSM within Nairobi. This approach will produce a measure of the of the population size of TSMSM, as perceived by TSMSM participating in the RDS study. This method is based on the assumption that members of the population have specialized knowledge on the population and that personal opinion expressed in private will not be influenced by others' responses (44). The estimate obtained will be examined using the median, mode, and mean responses and compared to the yields of other PSE methods.

#### Successive Sampling-RDS

This is a novel PSE method proposed by Handcock et al. (68). As such, the method is also referred to as Handcock-RDS or sequential sampling-RDS. It is based on the assumption that during RDS, participants with larger network sizes are more likely to be recruited earlier than those with smaller network sizes. Over the recruitment period, with SS without replacement, the probability of being sampled over time is proportional to the network size of the remaining members of the population. The SS-RDS method uses self-reported individual network size (i.e., the number of other members of the target population an individual participant knows) as the informative measure of the target population. The SS-RDS method uses a prior estimate in combination with the specified distribution and the data (the self-reported network size in RDS) to calculate the posterior PSE, through a Bayesian approach. In this study, the PSE obtained from the “wisdom of the crowds” method will be used as the prior estimate, and then the posterior PSE calculated using the RDS-A/RDSAT software.

#### RDS—Network Based Capture Recapture

This is a novel method proposed by Dombrowski et al. (69). In this method, study participants will be asked to provide some personal characteristics (approximate height, approximate weight, ethnicity, and handedness) and a “telefunken code. A “telefunken code” will be derived from the last four digits of participants' mobile phone number. The code is created where phone digits are coded as odd or even and low (0–4) or high (5–9)^1^. The participants will then be asked to randomly select three TSMSM contacts from their mobile phone directory. A participant will then be asked about the randomly selected contacts, in order to obtain data on the contacts' personal characteristics and “telefunken code.” The “telefunken code” together with the approximate height, approximate weight, ethnicity, and handedness will produce an almost unique anonymous code for each participant. This will facilitate the matching of the participant to contacts described by another participant's interview. For the purpose of PSE, study participants will be treated as the “capture” population, while each of the contacts provided during the interviews (“reports”) will be considered a “recapture assay.” The number of the original participants discovered via recapture assays (as a proportion of the total number of assays), will be used as a basis for estimating the overall size of the TSMSM population. The following formula and parameters will be used for PSE:


$$\begin{array}{l} N=n\;*\;\frac{s}{r} \end{array}$$

where:

*N* = TSMSM PSE,
*n* = Number of MSM captured in the survey with valid “telefunken codes”,
*s* = Number of valid “telefunken codes” reported by TSMSM in the study,
*r* = Excluding false matches, number of TSMSM' “telefunken codes” mentioned by other MSM.

### Phase III: In-depth Interviews With TSMSM and FGD With HCP Working in Tertiary Institutions

The data collected in this phase will be used to reach the second and third aims of the study. In-depth interviews with 20 TSMSM selected from the 200 in the IBBA will be held separately with each of the participants, in a private room at the PHDA study office. The aims of the interviews will be to explore TSMSM's experiences with access and utilization of health services and their self-identified strategies for improving these services. An interview guide with open ended questions will be used for this purpose.

Selected HCP working in tertiary institutions who will have been previously contacted will be invited to a central location such as the boardroom of the study office for the FGD. This will happen after the IBBA so that participating TSMSM do not get to meet HCP from their institutions. Alternatively, the discussions could take place in another central location as preferred by the HCP. The aim of the FGD is to assess the knowledge, attitudes, and practices of HCP toward provision of services to TSMSM. A discussion guide with questions covering the various aspects to be assessed will be used. The interview/discussion guides that will be used in this phase were developed for this study.

### Data Management

#### Qualitative Data Management

Each interview in phases I and III will be conducted by an interviewer who will also take notes. Focus group discussions will be led by a facilitator (the PI) assisted by a note-taker. Both the interviews and FGD will be audio-recorded and thereafter transcribed. In addition to note-taking being part of best practice for qualitative research (70), notes are also a backup to the audio-recordings in case the latter are spoilt or lost before transcription. Transcription and translation where needed, will be done by a member of the study team. The hard copies of the notes taken and printouts of the transcripts will be stored in locked file cabinets, in locked offices and access limited to authorized study staff only. The audio-recordings will be transferred from the recorder to a password protected hard drive and project computer. Thereafter, the recordings will be deleted from the recorders. The soft copies of the transcription will also be stored in a password protected computer. After transcription, a member of the study team will read the transcripts as he/she listens to the audio-recording to ensure that the transcripts and translations if any, are accurate.

#### Quantitative Data Management

During the IBBA in phase II, participants will electronically complete the behavioral questionnaire and the data will automatically be saved in a database on REDCap hosted at the University of the Witwatersrand, Johannesburg, South Africa. To assure quality of data, built in checks will be programmed into the REDCap control file, to be able to verify completeness and internal consistency of data automatically. Skip patterns or branching logic will be programmed in the questionnaire to ensure each participant is asked appropriate questions during the self-interview. Different scenarios with skip patterns will be thoroughly pre-tested to ensure correctness and consistency before the actual data collection commences. For the PSE questionnaires which will be interviewer-administered, the data will also be entered into a REDCap database developed for the study. To ensure the quality of data, the project coordinator will conduct checks for every 10th interview—both for IBBA and PSE—to verify for completeness and internal consistency. Both databases will be password protected and only accessed by research team members authorized by the PI. Hard copies of completed PSE questionnaires will be filed and kept in a locked cabinet. Only unique study codes, and not identifiable information will be used on the questionnaires. Unique study codes from the survey and HIV/STI testing will be managed by the project coordinator.

Coupon management to check validity, track distribution, and monitor reimbursements will be done using an Excel RDS Coupon Management File, 2010 (NGO Iskorak, Zagreb, Croatia). NetDraw v 2.170 (Analytic Technologies) will be used to monitor weekly reports of participant characteristics and recruitment chain graphs. This will help the study team make adjustments to coupon distribution so as to manage recruitment and maximize representativeness of the study sample. Initially, participants will be issued with three coupons to recruit their peers. This will be tapered off to two, one, and then zero coupons as the calculated sample size is approached and eventually reached. The following information will be displayed on the coupons: name of the study without disclosing same-sex behavior (Project Bespoke: health study for college/university male students), physical address of the study site with a map, telephone contact, hours of operation, coupon number, dates of coupon issue, and expiration, instructions to recipients on how to issue coupons, and brief description of the services that will be offered during the study.

### Data Analysis

#### Qualitative Data Analysis

Transcripts will be entered into NVivo software version 11 (QSR International) for coding and analysis. A thematic framework approach will be adopted. Both deductive (set a priori) and inductive (emerging from the data) themes will be analyzed. Themes will be supported with verbatim excerpts from the transcripts. To enhance trustworthiness of the data, coding will be done independently by two members of the study team, who will then compare codes for agreement and decide whether to merge some codes, get rid of others or come up with new ones.

#### Quantitative Data Analysis

As per recommendations (71), specialized analyses will be carried out using RDS-Analyst (RDS-A) software (University of California—Los Angeles) or RDS Analysis Tool (RDSAT) software (Cornell University). These analyses will produce population prevalence estimates of HIV/STI, and confidence intervals of variables, adjusting for unequal probabilities of inclusion due to varying social network sizes and homophily (the similarities in characteristics of persons within their social networks). The resulting data will then be exported to STATA software version 15 (StataCorp LLC) for further analysis. The primary analyses will be the adjusted population estimates of disease prevalence (HIV/STI), key risk behaviors (e.g., unprotected sex, multiple sexual partners, UAI), and access to and use of HIV/STI prevention, care and treatment services. Categorical variables will be described using frequencies. Normally distributed continuous variables will be described using means (with standard deviations) and non-normally distributed continuous variables will be described using medians (with IQR). Bi-variate analysis of variables will be done using Chi-square for categorical variables and Student's *t*-test for continuous variables. Finally, a multivariate analysis of variables associated with HIV/STI infection will be done using logistic (or binomial) regression. Population size estimation will be calculated using wisdom of the crowds, SS-RDS, and RDS-network based capture-recapture methods.

## Discussion

To the best of our knowledge, this is the first study in SSA to deploy RDS in conducting an IBBA of HIV/STI among TSMSM. Data from this study will be useful in informing HIV/STI prevention programming for this key and vulnerable population, by the ministries of health and education, tertiary education institutions, CBO/NGO, advocacy groups, and other interested stakeholders. One of the uniqueness of this study is the incorporation of supplemental qualitative components—formative research, in-depth interviews with TSMSM, and FGD with HCP—that seek to lay the ground for, and provide a deeper meaning of the quantitative data obtained from the IBBA, respectively. In this way, it is possible to obtain deeper perspective from both TSMSM and HCP, and thus begin to understand how to best design interventions for HIV/STI prevention as well as other healthcare services for TSMSM. The other strength of this study is the inclusion of the PSE section in the IBBA. Data on PSE of TSMSM will be useful in advocating for appropriate resource allocation toward health interventions for this population. Use of computer-formatted interviews on the REDCap software also constitutes other strengths of the study, including: consistency in the way questions are asked thus maximizing standardization; limiting handling of data forms; protecting participant confidentiality; and direct data capturing thereby decreasing staff effort and enhancing data quality.

Tertiary student men who have sex with men who are the primary participants in the study are a hard-to-reach population without an obvious sampling frame, due to criminalization of same-sex behavior in Kenya (72). This will be mitigated by using RDS, which is both a sampling and recruitment method for hidden populations. In addition, the study may run the risk of not reaching the required sample size due to refusal to participate and dropping out. The formative phase of the study will be used to engage the community of service providers and TSMSM, so as to build support for subsequent phases of the study and help attain the required sample size. Furthermore, as most data in the behavioral survey for TSMSM will be self-reported, this may present various forms of bias in the study. Recall bias may occur since participants will be asked to report things that happened to them in the past. To mitigate this, the time-frame of experiences in the survey has been limited to the last 12 months in most cases, last 6 or 3 months in some cases, and only a few questions ask about life-time experiences. Additionally, since the survey has questions on topics such as sexual behavior, alcohol and drug use, participants may underreport on these topics resulting in social desirability bias. This will be mitigated by using self-interviews as compared to interviewer-administered questionnaires, where the propensity to underreport is increased with the latter.

## Ethics and Dissemination

### Informed Consent

Participants will be provided with information about the study, including: their roles in the study; general nature of interview questions; procedures for specimen collection and testing and duration and frequency of participation. Participants will then be given opportunities to ask questions and/ or seek clarifications about the study. Thereafter, participants will provide written informed consent to participate in the study.

### Age of Participants

The study will recruit TSMSM who are at least 18 years old. Tertiary student men who have sex with men below 18 years of age will be ineligible to participate. Staff working in CBO/NGO and HCP will also be at least 18 years old.

### Protection of Study Participants

A major ethical concern of this study is that taking part may reveal that participants are engaging in same-sex sexual activity which is both illegal and stigmatized in Kenya. Unintended disclosure of personal information collected from the study may expose participants to discrimination, stigmatization, and potential harm. Several measures will be taken to minimize the risk of information disclosure, including: study staff will check recruitment coupons to ensure their validity and use an interviewer administered questionnaire to screen prospective participants for study eligibility; no identifying information will be written on study documents nor laboratory specimens associated with a participant; all paper-based study materials will be stored in locked file cabinets, in locked offices and access limited to authorized study staff; audio-transcripts will be stored in a password protected computer accessible only to authorized study staff; access to study databases will be limited to authorized study staff only; and same-sex sexual behavior will not be reported to authorities. All staff working with participants will be trained on the importance of confidentiality and be required to sign a confidentiality agreement. In addition, the proposed staff for this project have worked with MSM in previous studies such as TRANSFORM and are already aware of the requirements of protection of participants.

A HIV/STI positive result may also subject a participant to psychological and emotional stress. To lessen these harms, trained, certified, and experienced counselors will provide pre- and post-test counseling to all participants. Moreover, since the study asks TSMSM questions on their experiences of stigma, discrimination and violence; mental health—depression, anxiety, and stress; and alcohol and drug use, a distress protocol has been developed. The protocol outlines the course of actions to be taken by the study counselor should a participant exhibit acute emotional distress, safety concern or imminent danger to himself or others, at any stage of the study.

Written informed consent forms for TSMSM may pose a risk of exposing their identity. This will be mitigated by: using unique survey codes to link the forms, data, and specimens; storing the informed consent forms securely and separately from data and biological specimens collected during and after the study; and limiting access to the informed consent forms to study staff members authorized by the PI. Furthermore, possession of the consent form might pose a safety threat to TSMSM by revealing their identity and to HCP by revealing that they took part in a study on a subject that is both criminalized and stigmatized in Kenya. For both TSMSM and HCP who feel that this may happen to them, the study team will provide them with minimal information (the contact information of the study team and relevant research ethics committees) and not the whole copy of the consent form. In addition, copies of the information and consent forms will be retained at the study office, where a participant can pick them when and if they are comfortable to do so.

Healthcare providers participating in the FGD where there is neither confidentiality nor anonymity may also face more risks. A participant could express an opinion which other participants do not like. Such a participant could be at risk of social harms such as being reported to the employer, religious leaders, and/or social groups. To minimize this, participants in the FGD will be reminded to respect the privacy of their fellow participants and not to repeat what is said in the FGD to other people outside the group. Though what is said outside the FGD cannot be completely censored, a non-disclosure statement will be included in the consent form and participants required to agree to it.

The study office is located on the fifth floor of a building in the central business district of the city and participants can walk in without raising any suspicion from members of the public. This study area has previously been used for other studies among MSM such as TRANSFORM. Furthermore, the Ministry of Health through National AIDS and STI Control Program (NASCOP), runs a Key Populations Program whose work supports research among key populations and guards against harassment by security agencies. NASCOP has issued a “to whom it may concern” letter to support this study.

### Potential Benefits

Study participants will be provided with the following services at the study site free of charge: counseling and testing for HIV/STI; treatment for STI; referral for HIV care and treatment at MSM-friendly health facilities or facilities of their choice; condoms and lubricants; and HIV/STI educational materials.

### Participant Compensation

Reimbursements will be offered to compensate participants in the IBBA for the significant level of effort and time required to complete the study activities, and cater for transport costs. Participants will be given a primary incentive of KES 1,000 (USD 10) for participating in the study and a secondary incentive of KES 300 (USD 3) for each participant recruited. Thus, a participant who recruits three other participants would get KES 1,900 (USD 19) in total as compensation. NGO/CBO staff and HCPs will also each be reimbursed KES 1,000 (USD 10) for time spend in the study and to cover transport costs.

### Ethical Review and Approval

This study protocol has been reviewed and approved by University of the Witwatersrand Human Research Ethics Committee-Medical (M200215) and University of Nairobi-Kenyatta National Hospital Ethics and Research Committee (P990/12/2019).

### Dissemination

The findings from this study will be disseminated through the Key Population Program which is led by NASCOP under the ministry of health. Community meetings will also be organized to share the findings with TSMSM community members. The findings will be also shared with both participating and other interested CBO/NGO that serve MSM in Kenya, as well as HCP working in tertiary institutions. Additionally, the findings will be disseminated through peer reviewed journals, presentations at conferences, engagements with stakeholders, and with policy makers in Kenya including the Kenyan ministries of health and education. Finally, a study closure report will be submitted to the research ethics committees in Kenya and South Africa.

## Ethics Statement

The studies involving human participants were reviewed and approved by University of the Witwatersrand Human Research Ethics Committee (Medical), Kenyatta National Hospital—University of Nairobi Ethics and Research Committee. The patients/participants provided their written informed consent to participate in this study.

## Author Contributions

SM, PM and TP-P conceptualized the study, designed the study protocol, and its instruments. SM acquired funding for the study and drafted the manuscript. PM and TP-P reviewed the manuscript. All authors have read and approved the final manuscript.

## Funding

SM was supported by the Consortium for Advanced Research Training in Africa (CARTA). CARTA is jointly led by the African Population and Health Research Center and the University of the Witwatersrand and funded by the Carnegie Corporation of New York (Grant No. G-19-57145), Sida (Grant No: 54100113), Uppsala Monitoring Centre and the DELTAS Africa Initiative (Grant No. 107768/Z/15/Z). The DELTAS Africa Initiative is an independent funding scheme of the African Academy of Sciences (AAS)'s Alliance for Accelerating Excellence in Science in Africa (AESA) and supported by the New Partnership for Africa's Development Planning and Coordinating Agency (NEPAD Agency) with funding from the Wellcome Trust (UK) and the UK government. The statements made and views expressed are solely the responsibility of the Fellow.

## Conflict of Interest

The authors declare that the research was conducted in the absence of any commercial or financial relationships that could be construed as a potential conflict of interest.

## Publisher's Note

All claims expressed in this article are solely those of the authors and do not necessarily represent those of their affiliated organizations, or those of the publisher, the editors and the reviewers. Any product that may be evaluated in this article, or claim that may be made by its manufacturer, is not guaranteed or endorsed by the publisher.

## Footnotes

^1^For instance, for a participant whose phone number last four digits are 1234, the “telefunken code” is: odd-even-odd-even-low-low-low-low.

## Abbreviations

AIDS: acquired immunodeficiency syndrome
CARTA: consortium for advanced research training in Africa
CBO: community-based organization
CT: *Chlamydia trachomatis*
HCP: healthcare providers
HIV: human immunodeficiency virus
IBBA: integrated bio-behavioral assessment
IQR: inter-quartile range
MG: *Mycoplasma genitalium*
MSM: men who have sex with men
NASCOP: national AIDS and STI control program
NG: *Neisseria gonorrhoeae*
NGO: non-governmental organization
PHDA: partners in health and development in Africa
PI: principal investigator
PSE: population size estimation
RDS: respondent-driven sampling
REDCap: research electronic data capture
SSA: Sub-Saharan Africa
STATA: software for statistics and data science
STI: sexually transmitted infections
TP: *Treponema pallidum*
TSMSM: tertiary student men who have sex with men
TV: *Trichomonas vaginalis*
UAI: unprotected anal intercourse
WHO: World Health Organization
YMSM: young men who have sex with men.
